# Supplementary figures and images for: The impact of COVID-19 pandemic course in the number and severity of hospitalizations for other natural causes in a large urban center in Brazil
Source: PLOS Glob Public Health. 2021 Dec 20;1(12):e0000054. doi: 10.1371/journal.pgph.0000054 (PMC10021898; doi:10.1371/journal.pgph.0000054)

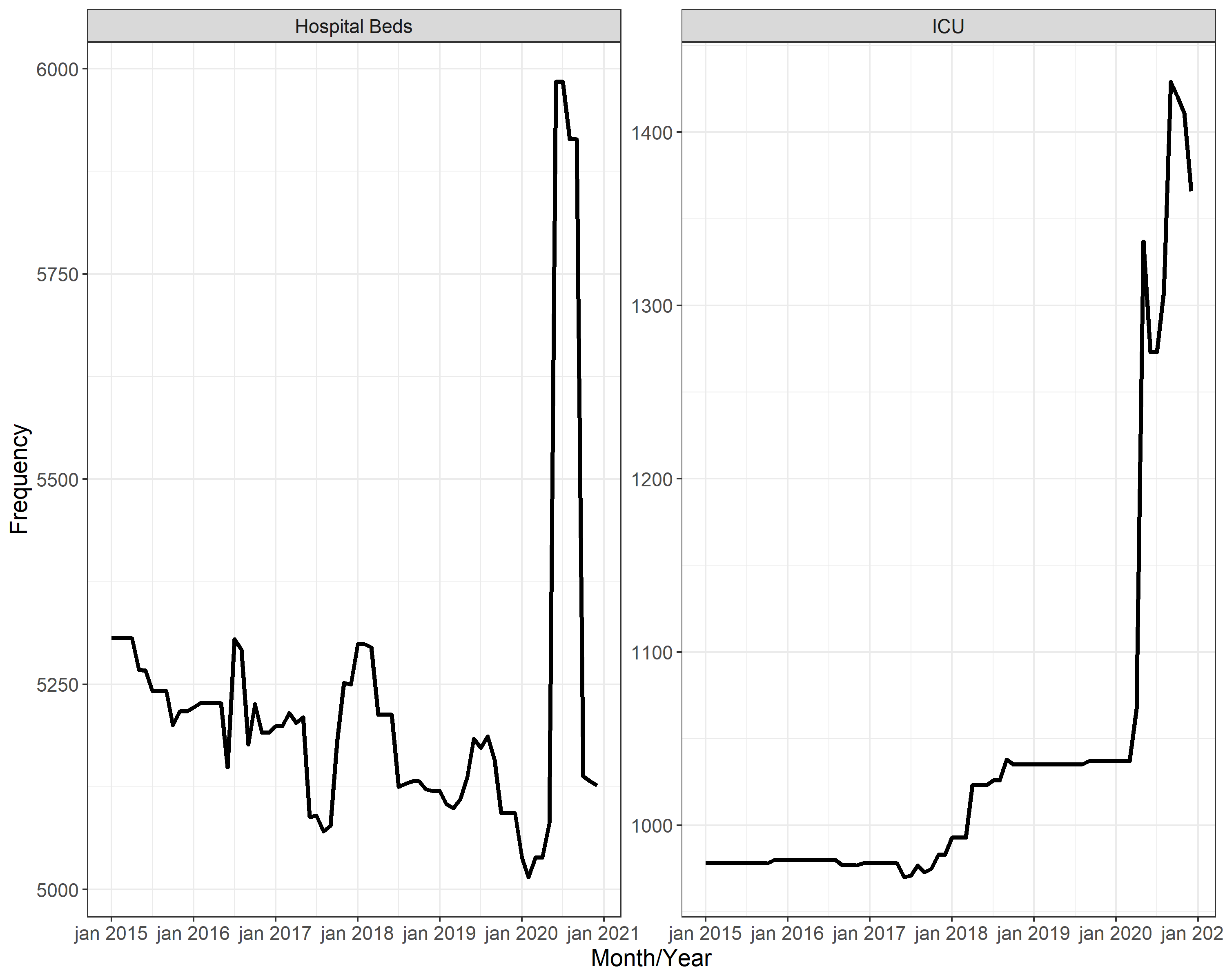

Supplement: S1 Fig — (PNG) [file pgph.0000054.s001.png]

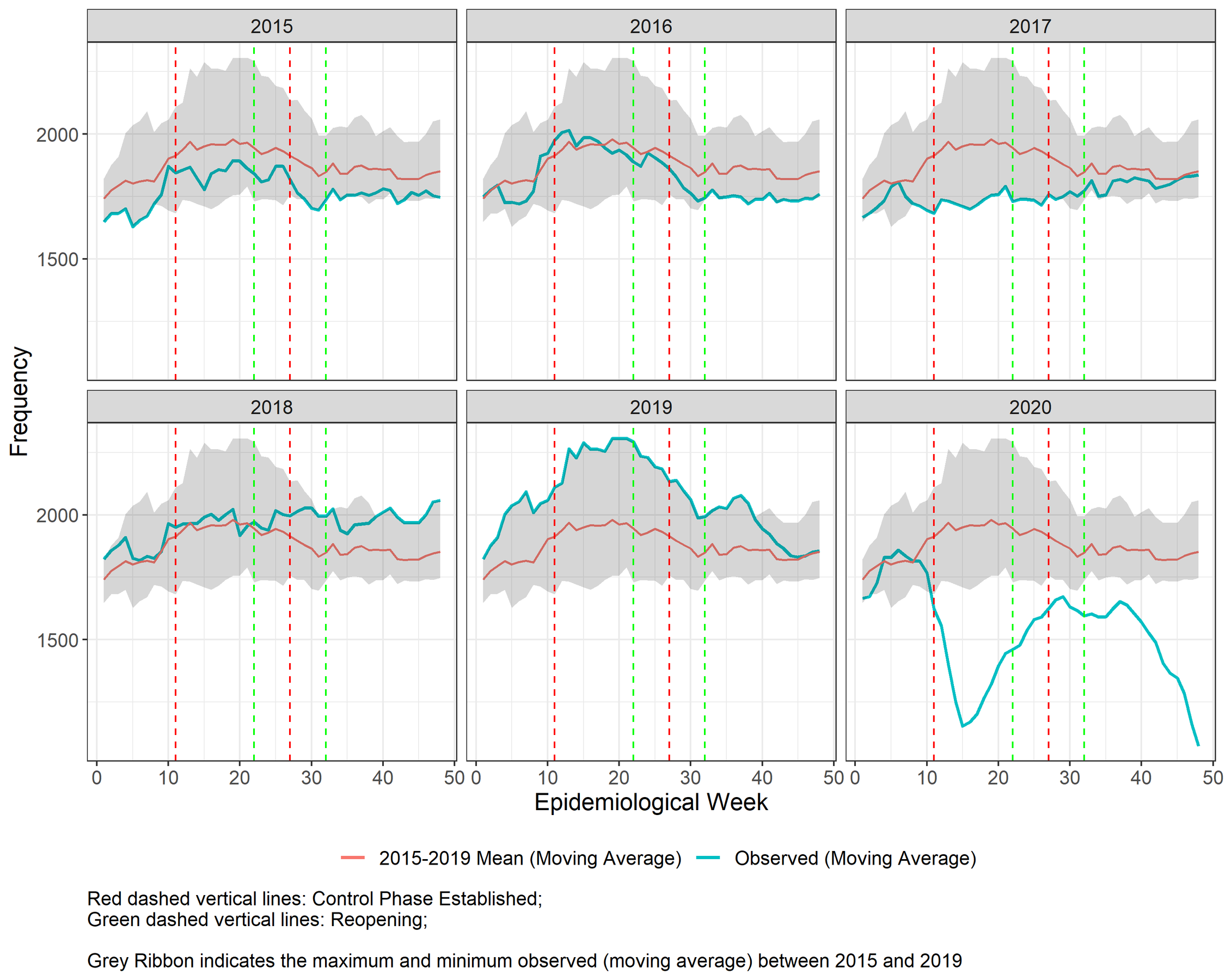

Supplement: S2 Fig — Five-week moving average of number of hospital admissions observed in Belo Horizonte in the epidemiological weeks (EW) 1 to 48, from 2015 to 2020 (blue lines), and the mean of the same EW of 2015–2019 (red lines) for all natural causes. Vertical lines refer to the weeks of control (red) and reopening phases (green). Grey ribbon indicates the maximum and minimum observer (moving average) for the period. (TIF) [file pgph.0000054.s002.tif]

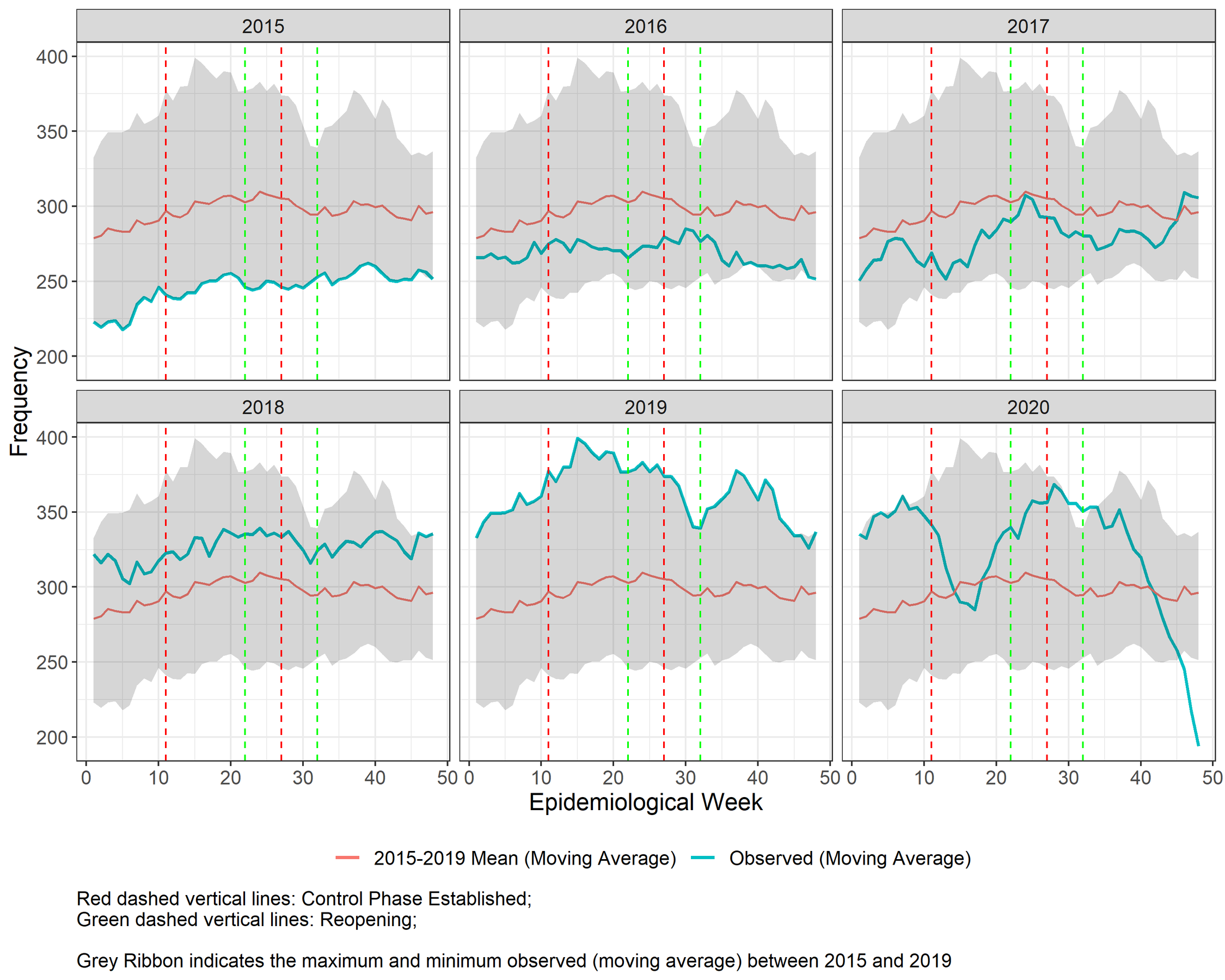

Supplement: S3 Fig — Five-week moving average of number of intensive care unit admissions observed in Belo Horizonte in the epidemiological weeks (EW) 1 to 48, from 2015 to 2020 (blue lines), and the mean of the same EW of 2015–2019 (red lines) for all natural causes. Vertical lines refer to the weeks of control (red) and reopening phases (green). Grey ribbon indicates the maximum and minimum observer (moving average) for the period. (TIF) [file pgph.0000054.s003.tif]
