# Supplementary material for: The impact of COVID-19 pandemic course in the number and severity of hospitalizations for other natural causes in a large urban center in Brazil
Source: PLOS Glob Public Health. 2021 Dec 20;1(12):e0000054. doi: 10.1371/journal.pgph.0000054 (PMC10021898; doi:10.1371/journal.pgph.0000054)
Supplement: S1 Table — (DOCX) [file pgph.0000054.s004.docx]

**S2 Table:** Hospital Admission Month and Hospitalization Processing Month in Belo Horizonte, from January 2019 to May 2020.

|  |  | Processing Month | | | | | | | | | | | | | | | | | |
| --- | --- | --- | --- | --- | --- | --- | --- | --- | --- | --- | --- | --- | --- | --- | --- | --- | --- | --- | --- |
|  |  | 2019-01 | 2019-02 | 2019-03 | 2019-04 | 2019-05 | 2019-06 | 2019-07 | 2019-08 | 2019-09 | 2019-10 | 2019-11 | 2019-12 | 2020-01 | 2020-02 | 2020-03 | 2020-04 | 2020-05 |  |
| Hospital Admission Month | 2019-01 | 32,32 | 41,01 | 16,58 | 8,25 | 1,24 | 0,36 | 0,06 | 0,02 | 0,02 | 0,02 | 0,02 | 0,01 | 0,01 | 0,01 | 0,01 | 0,01 | 0,01 |  |
|  | 2019-02 | 0 | 33,1 | 43,07 | 14,85 | 7,32 | 1,33 | 0,29 | 0,03 | 0 | 0,01 | 0 | 0 | 0 | 0 | 0 | 0 | 0 |  |
|  | 2019-03 | 0 | 0 | 33,81 | 40,45 | 16,94 | 7,15 | 1,23 | 0,32 | 0,07 | 0,02 | 0,02 | 0 | 0 | 0 | 0 | 0 | 0 |  |
|  | 2019-04 | 0 | 0 | 0 | 37,23 | 37,78 | 16,01 | 7,54 | 1,01 | 0,32 | 0,05 | 0,01 | 0,01 | 0,01 | 0,01 | 0 | 0,01 | 0,01 |  |
|  | 2019-05 | 0 | 0 | 0 | 0 | 35,97 | 38,93 | 16,02 | 7,43 | 1,19 | 0,3 | 0,04 | 0,01 | 0,01 | 0,01 | 0,01 | 0,01 | 0,01 |  |
|  | 2019-06 | 0 | 0 | 0 | 0 | 0 | 32,36 | 45,87 | 13,17 | 6,98 | 1,25 | 0,34 | 0,03 | 0 | 0 | 0 | 0 | 0 |  |
|  | 2019-07 | 0 | 0 | 0 | 0 | 0 | 0 | 37,18 | 42,31 | 12,08 | 6,87 | 1,07 | 0,39 | 0,09 | 0,02 | 0,01 | 0 | 0 |  |
|  | 2019-08 | 0 | 0 | 0 | 0 | 0 | 0 | 0 | 37,62 | 41,18 | 12,54 | 6,46 | 1,58 | 0,44 | 0,07 | 0,03 | 0,02 | 0,02 |  |
|  | 2019-09 | 0 | 0 | 0 | 0 | 0 | 0 | 0 | 0 | 35,65 | 44,73 | 9,96 | 7,8 | 1,39 | 0,39 | 0,06 | 0,01 | 0 |  |
|  | 2019-10 | 0 | 0 | 0 | 0 | 0 | 0 | 0 | 0 | 0 | 39,51 | 36,19 | 12,6 | 9,99 | 1,13 | 0,49 | 0,06 | 0,01 |  |
|  | 2019-11 | 0 | 0 | 0 | 0 | 0 | 0 | 0 | 0 | 0 | 0 | 37,76 | 34,52 | 17,56 | 8,39 | 1,33 | 0,37 | 0,06 |  |
|  | 2019-12 | 0 | 0 | 0 | 0 | 0 | 0 | 0 | 0 | 0 | 0 | 0 | 36,4 | 34,17 | 20,21 | 7,75 | 1,09 | 0,28 |  |
