# Supplementary material for: The impact of COVID-19 pandemic course in the number and severity of hospitalizations for other natural causes in a large urban center in Brazil
Source: PLOS Glob Public Health. 2021 Dec 20;1(12):e0000054. doi: 10.1371/journal.pgph.0000054 (PMC10021898; doi:10.1371/journal.pgph.0000054)
Supplement: S2 Table — (DOCX) [file pgph.0000054.s005.docx]

**S6 Table.** Mean and standard deviation (in parentheses) of the duration of hospital stay in days for selected groups of diseases, in Belo Horizonte, from 2015 to 2019 and in epidemiological weeks 9-48 of 2020.

| Group of diseases | 2015 | 2016 | 2017 | 2018 | 2019 | **2015-**  **2019** | 2020 |  |
| --- | --- | --- | --- | --- | --- | --- | --- | --- |
| Non-COVID-19 Natural Causes | 7.19  (10.1) | 7.21  (10) | 7.36  (10.09) | 7.05  (9.39) | 6.61  (8.6) | 7.07  (9.62) | 6.85  (7.94) |  |
| Infectious diseases | 15.41  (16.35) | 13.39  (15.4) | 16.36  (16.05) | 15.68  (15.13) | 11.25  (12.49) | 14.07  (15.04) | 12.53  (11.34) |  |
| Neoplasms | 6.1  (7.63) | 6.15  (7.76) | 5.89  (7.28) | 6.15  (7.48) | 5.59  (6.6) | 5.97  (7.35) | 5.36  (6.01) |  |
| Circulatory system | 7.75  (9.87) | 7.83  (9.29) | 8.91  (10.01) | 8.85  (9.27) | 8.44  (8.37) | 8.34  (9.37) | 8.19  (7.68) |  |
| Respiratory diseases | 7.41  (10.25) | 7.88  (11) | 7.94  (11.15) | 7.67  (9.42) | 7.58  (9.45) | 7.69  (10.25) | 7.19  (8.17) |  |
