# Supplementary material for: The impact of COVID-19 pandemic course in the number and severity of hospitalizations for other natural causes in a large urban center in Brazil
Source: PLOS Glob Public Health. 2021 Dec 20;1(12):e0000054. doi: 10.1371/journal.pgph.0000054 (PMC10021898; doi:10.1371/journal.pgph.0000054)
Supplement: S3 Table — (DOCX) [file pgph.0000054.s006.docx]

**S7 Table.** Difference in the number of hospitalization, and number and proportion of intensive care unit admission and in-hospital deaths from epidemiological weeks (EW) 9-48, 2020 (observed), and the 2015-2019 mean for the same EW, according to sex, in Belo Horizonte.

| **Variables** | **Female** | | | **Male** | | |
| --- | --- | --- | --- | --- | --- | --- |
|  | **2020** | **2015-2019*^1^*** | **Difference** | **2020** | **2015-2019*^1^*** | **Difference** |
| **Hospital admissions (HA)^2^** | | | | | | |
| Non-COVID-19 natural causes | 26869 | 38777 (34692;43482) | -11908* | 27853 | 37063 (33080;42197) | -9210* |
| Infectious diseases | 2504 | 2944 (2036;4547) | -440* | 3095 | 3777 (2854;5196) | -682* |
| Neoplasms^2^ | 4374 | 6017 (5222;6788) | -1643* | 4019 | 4872 (4206;5569) | -853* |
| Cardiovascular diseases | 4512 | 6484 (5592;7581) | -1972* | 4947 | 5954 (5112;6964) | -1007* |
| Respiratory diseases | 3501 | 4695 (3923;5492) | -1194* | 3856 | 5108 (4322;6067) | -1252* |
| **ICU admissions^2^** | | | | | | |
| Non-COVID-19 natural causes | 5001 | 5428 (4337;6749) | -427* | 6167 | 6584 (5336;8121) | -417* |
| Infectious diseases | 983 | 886 (591;1239) | 97* | 1240 | 1152 (807;1590) | 88 |
| Neoplasms^2^ | 636 | 724 (525;935) | -88* | 609 | 734 (512;972) | -125* |
| Cardiovascular diseases | 1563 | 1777 (1361;2226) | -214* | 2038 | 2296 (1740;2878) | -258* |
| Respiratory diseases | 572 | 556 (336;803) | 16 | 704 | 675 (420;954) | 29 |
| **% ICU admissions^3^** | | | | | | |
| Non-COVID-19 natural causes | 18.8 | 14.0 (11.9;16.3) | 4.8* | 22.1 | 17.7 (15.4.20.2) | 4.4* |
| Infectious diseases | 39.3 | 31.9 (21.9;41.2) | 7.4* | 39.4 | 31.2 (23.5;39.4) | 8.2* |
| Neoplasms | 14.4 | 12.1 (9.1;15.3) | 2.3* | 14.9 | 15.0 (11.2;18.8) | -0.1 |
| Cardiovascular diseases | 34.7 | 27.9 (20.0;35.8) | 6.8* | 41.2 | 38.5 (31.9;44.9) | 2.7* |
| Respiratory diseases | 16.7 | 11.9 (7.5;16.3) | 4.8* | 18.4 | 13.3 (08.9;18.1) | 5.1* |
| **In-Hospital deaths^2^** | | | | | | |
| Non-COVID-19 natural causes | 1730 | 1905 (1582;2306) | -175* | 1831 | 2098 (1700;2591) | -267* |
| Infectious diseases | 608 | 534 (354;741) | 74* | 685 | 636 (442;895) | 49 |
| Neoplasms | 318 | 437 (288;603) | -119* | 282 | 465 (301;636) | -183* |
| Cardiovascular diseases | 294 | 340 (217;475) | -46* | 294 | 329 189;487) | -35 |
| Respiratory diseases | 225 | 234 (123;368) | -9.4 | 228 | 246 (120;389) | -18 |
| **% In-Hospital deaths^3^** | | | | | | |
| Non-COVID-19 natural causes | 6.5 | 4.9 (4.0;5.9) | 1.6* | 6.6 | 5.7 (4.6;6.8) | 0.9* |
| Infectious diseases | 24.1 | 19.5 (12.3;26.9) | 4.6* | 22.1 | 17.4 (12.2;23.7) | 4.7* |
| Neoplasms | 7.4 | 7.3 (4.7;0.10.1) | 0.1 | 7.0 | 9.7 (6.1;13.5) | -2.7* |
| Cardiovascular diseases | 6.6 | 5.3 (3.3;7.5) | 1.3* | 5.9 | 5.5 (3.3;7.9) | 0.4 |
| Respiratory diseases | 6.4 | 5.1 (2.6;7.8) | 1.3* | 6.0 | 4.9 (2.4;7.6) | 1.1 |

*^1^*2015-2019 mean. In parenthesis, lower limit aggregates the lowest values for the same EW between 2015-2019 and the upper limit the aggregates the highest.

* p≤0.05.

*^2^*Sum of the observed values EW 10-48.

*^3^*Mean of the observed values EW 10-48.

*P-Value lower than 0.05.
