# Supplementary material for: The impact of COVID-19 pandemic course in the number and severity of hospitalizations for other natural causes in a large urban center in Brazil
Source: PLOS Glob Public Health. 2021 Dec 20;1(12):e0000054. doi: 10.1371/journal.pgph.0000054 (PMC10021898; doi:10.1371/journal.pgph.0000054)
Supplement: S4 Table — (DOCX) [file pgph.0000054.s007.docx]

**S8 Table.** Difference in the number of hospitalization, and number and proportion of intensive care unit admission and in-hospital deaths from epidemiological weeks (EW) 9-48, 2020 (observed), and the 2015-2019 mean for the same EW, according to age groups, in Belo Horizonte.

| **Variables** | |  | **0-29** | | | **30-59** | | | **60+** | | | |
| --- | --- | --- | --- | --- | --- | --- | --- | --- | --- | --- | --- | --- |
|  |  |  | **2020** | **2015-2019*^1^*** | **Difference** | **2020** | **2015-2019*^1^*** | **Difference** | **2020** | **2015-2019*^1^*** | **Difference** | |
|  | **Hospitalizations^2^** | | | | | | | | | | |  |
| Non-COVID-19 natural causes | |  | 9194 | 16528 (14681;18231) | -7334* | 21307 | 29876 (26549;33589) | -8569* | 24221 | 29436 (24898;34764) | -5215* | |
| Infectious diseases | |  | 1047 | 1997 (1364;2984) | -951* | 1970 | 2389 (1674;3554) | -419* | 2582 | 2334 (1562;3490) | 248* | |
| Neoplasms | |  | 553 | 857 (642;1074) | -304* | 3642 | 4771 (4058;5418) | -1129* | 4198 | 5262 (4568;5976) | -1064* | |
| Cardiovascular diseases | |  | 321 | 512 (309;749) | -191* | 3243 | 4837 (3899;5935) | -1594* | 5895 | 7089 (5985;8188) | -1194* | |
| Respiratory diseases | |  | 2043 | 4803(4029;5969) | -2760* | 2086 | 1661 (1305;2014) | 425* | 3228 | 3339 (2619;4104) | -111 | |
|  | **ICU admissions^2^** | | | | | | | | | | |  |
| Non-COVID-19 natural causes | |  | 1374 | 1726 (1380;2112) | -352* | 3803 | 4007 (3319;4868) | -204 | 5991 | 6278 (4779;8012) | -287 | |
| Infectious diseases | |  | 337 | 434 (260;641) | -97* | 671 | 598 (368;855) | 73. | 1215 | 1006 (653;1455) | 209* | |
| Neoplasms | |  | 71 | 97 (34;173) | -27* | 444 | 525 (344;723) | -81* | 730 | 837 (617;1084) | -107* | |
| Cardiovascular diseases | |  | 96 | 106 (37;179) | -10 | 1262 | 1452 (1141;1793) | -190* | 2243 | 2514 (1817;3209) | -271* | |
| Respiratory diseases | |  | 274 | 383 (229;554) | -109* | 406 | 296 (161;455) | 110* | 596 | 552 (315;824) | 44 | |
|  | **% ICU admissions^3^** | | | | | | | | | | |  |
| Non-COVID-19 natural causes | |  | 15.0 | 10.5 (8.5;12.9) | 4.5* | 17.9 | 13.4 (11.4;15.6) | 4.5* | 24.8 | 21.2 (18.3;24.1) | 3.6* | |
| Infectious diseases | |  | 31.1 | 22.8 (13.9;31.8) | 8.3* | 33.9 | 26.1 (17.9;34.6) | 7.8* | 46.7 | 44.1 (32.9;55.3) | 2.6 | |
| Neoplasms | |  | 13.0 | 11.2 (4.0;20.1) | 1.8 | 12.0 | 11.0 (7.4;14.8) | 1.0 | 17.3 | 15.9 (12.4;19.7) | 1.4 | |
| Cardiovascular diseases | |  | 31.1 | 21.9 (6.9;37.9) | 9.2* | 38.8 | 30.8 (21.7;39.9) | 8.0* | 38.2 | 35.3 (28.6;41.7) | 2.9* | |
| Respiratory diseases | |  | 13.8 | 8.2 (4.6;12.1) | 5.6* | 19.9 | 17.8 (10.5;26.4) | 2.1 | 18.5 | 16.3 (10.9;22.0) | 2.2 | |
|  | **In-Hospital deaths^2^** | | | | | | | | | | |  |
| Non-COVID-19 natural causes | |  | 120 | 185 (97;289) | -65* | 856 | 1004 (752;1281) | -148* | 2585 | 2814 (2352;3395) | -229 | |
| Infectious diseases | |  | 36 | 56 (11;110) | -20* | 307 | 289 (151;439) | 18 | 950 | 824 (574;1112) | 126* | |
| Neoplasms | |  | 12 | 23 (0;61) | -11* | 195 | 305 (171;447) | -110* | 393 | 575 (385;761) | -182* | |
| Cardiovascular diseases | |  | 7 | 17 (0;48) | -10* | 127 | 155 (80;243) | -28* | 454 | 496 (324;688) | -42* | |
| Respiratory diseases | |  | 20 | 26 (0;66) | -6* | 73 | 64 (16;122) | 9 | 360 | 389 (224;576) | -29 | |
|  | **% In-Hospital deaths^3^** | | | | | | | | | | |  |
| Non-COVID-19 natural causes | |  | 1.4 | 1.1 (0.6;1.8) | 0.3 | 4.0 | 3.4 (2.5;4.3) | 0.6* | 10.7 | 9.6 (8.2;11.1) | 1.1 | |
| Infectious diseases | |  | 3.3 | 3.0 (0.5;6.4) | 0.3 | 15.6 | 0.128 (6.6;18.7) | 2.8* | 36.6 | 36.7 (26.8;47.2) | 0.1 | |
| Neoplasms | |  | 2.2 | 2.6 (0.1;7.4) | -0.4* | 5.4 | 6.5 (3.7;9.8) | -1.1* | 9.3 | 11.0 (7.5;14.6) | -1.7* | |
| Cardiovascular diseases | |  | 2.3 | 3.7 (0.1;11.5) | -1.4* | 3.9 | 3.3 (1.6;5.3) | 0.6 | 7.8 | 7.0 (4.7;9.2) | 0.8 | |
| Respiratory diseases | |  | 1.1 | 0.6 (0.1;1.4) | 0.5 | 3.5 | 3.9 (0.9;7.4) | -0.4 | 11.1 | 11.7 (7.3;16.8) | -0.6 | |

*^1^*2015-2019 mean. In parenthesis, lower limit aggregates the lowest values for the same EW between 2015-2019 and the upper limit the aggregates the highest.

*^2^*Sum of the observed values EW 10-48. Difference in the number of hospitalization, and number and proportion of intensive care unit admission and in-hospital deaths from epidemiological weeks 9-48, 2020 (observed), and the 2015-2019 mean for the same EW, according to age groups, in Belo Horizonte.

*^3^*Mean of the observed values EW 10-48.

*P-Value lower than 0.05.
