# Supplementary material for: The impact of COVID-19 pandemic course in the number and severity of hospitalizations for other natural causes in a large urban center in Brazil
Source: PLOS Glob Public Health. 2021 Dec 20;1(12):e0000054. doi: 10.1371/journal.pgph.0000054 (PMC10021898; doi:10.1371/journal.pgph.0000054)

**S3 Text.** Tutorial to access DATASUS data used in the present analysis.

This is tutorial to help reaching the files with the data we have used. However, it is important to emphasize that the dataset we have used is part of the same information system, but it has sensitive information, and as such we were unable to share as we used it.

1. Access DATASUS webpage where the data is storaged.

<https://datasus.saude.gov.br/transferencia-de-arquivos/>


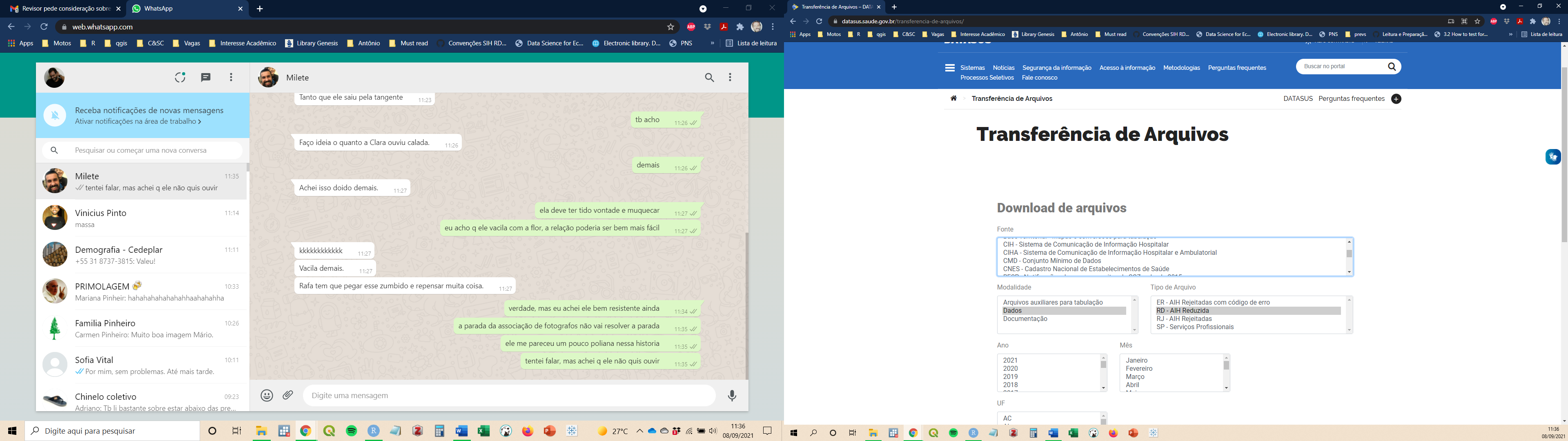


1. In the box named ‘Download de arquivos’, select the option

‘SIHSUS – Sistema de Informações Hospitalares’


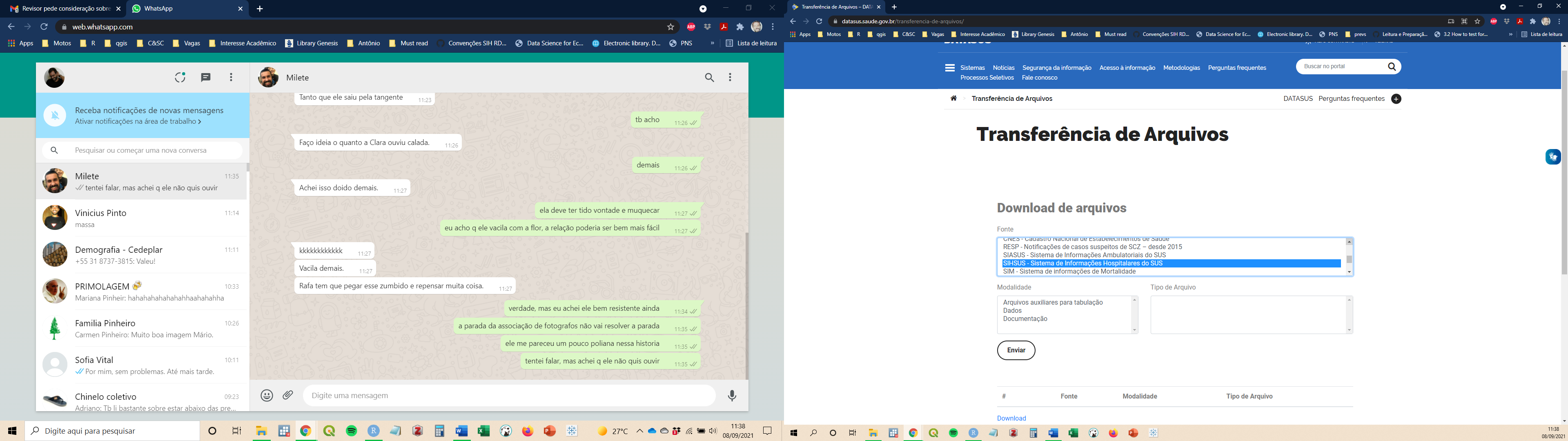


1. In the box named ‘Modalidade’, select the option ‘Dados’


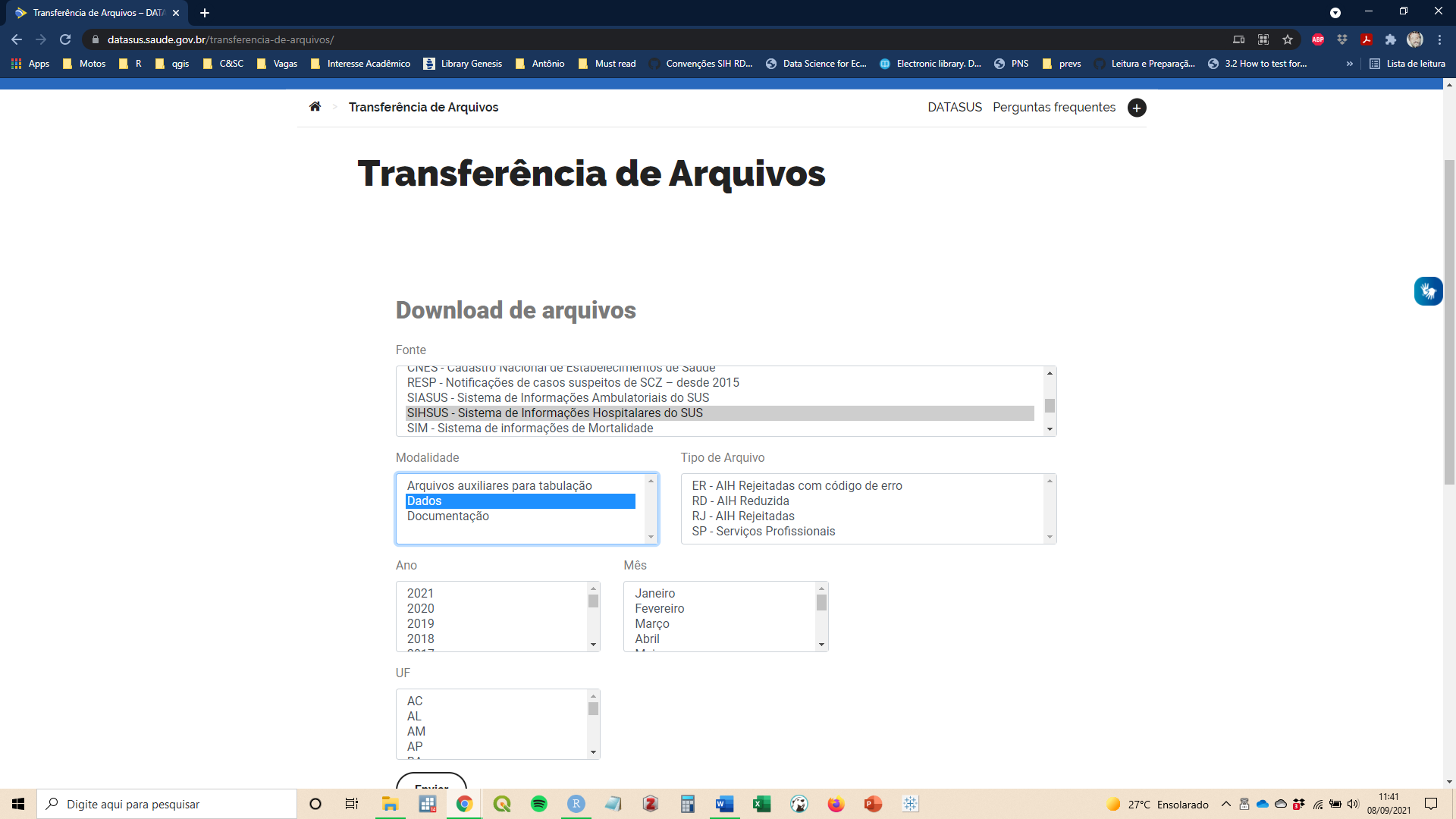


1. In the box named ‘Tipo de Arquivo’, select the option ‘RD – AIH Reduzida’


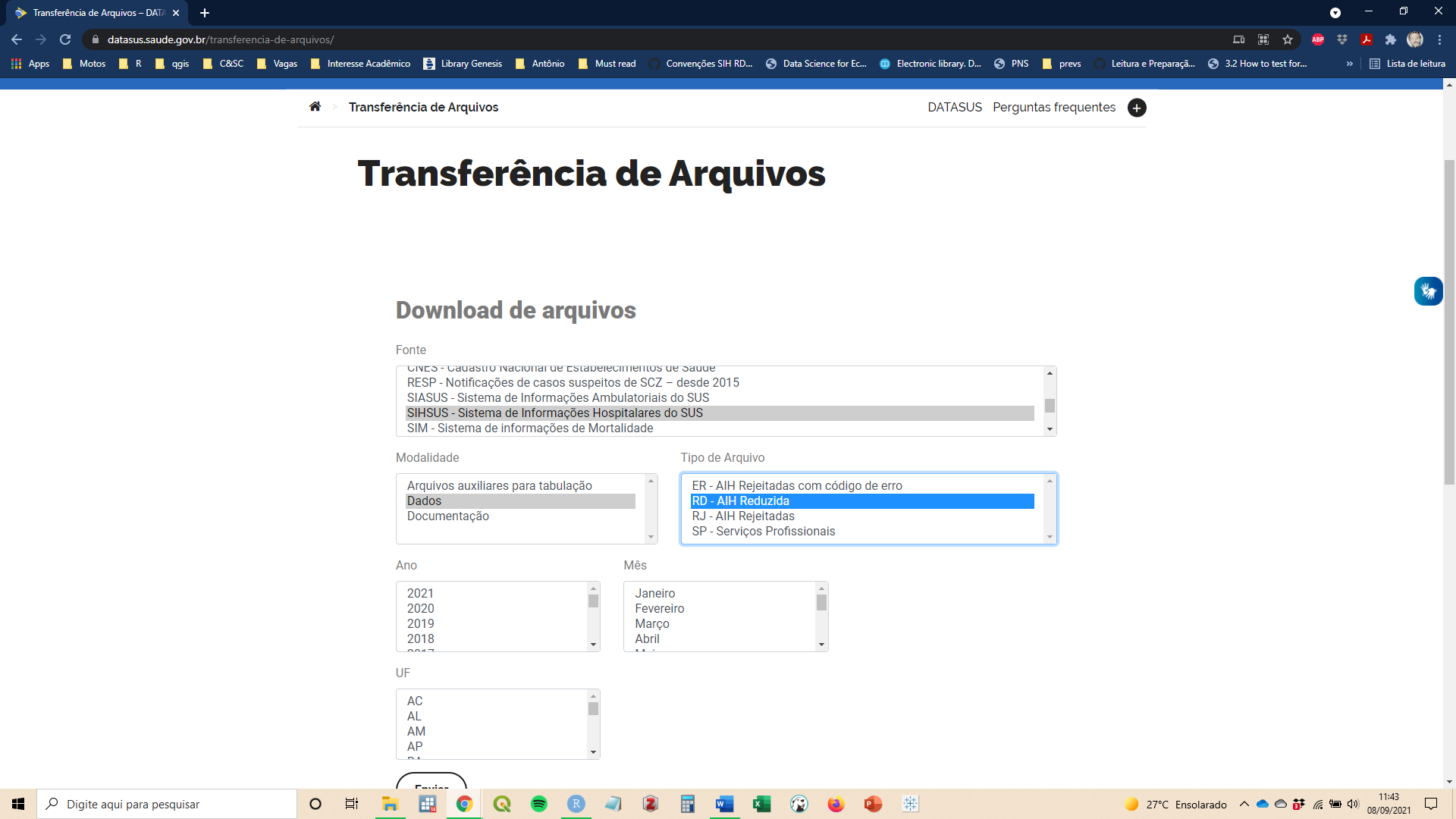


1. In the box named ‘Ano’, select the year or set of years of interest.


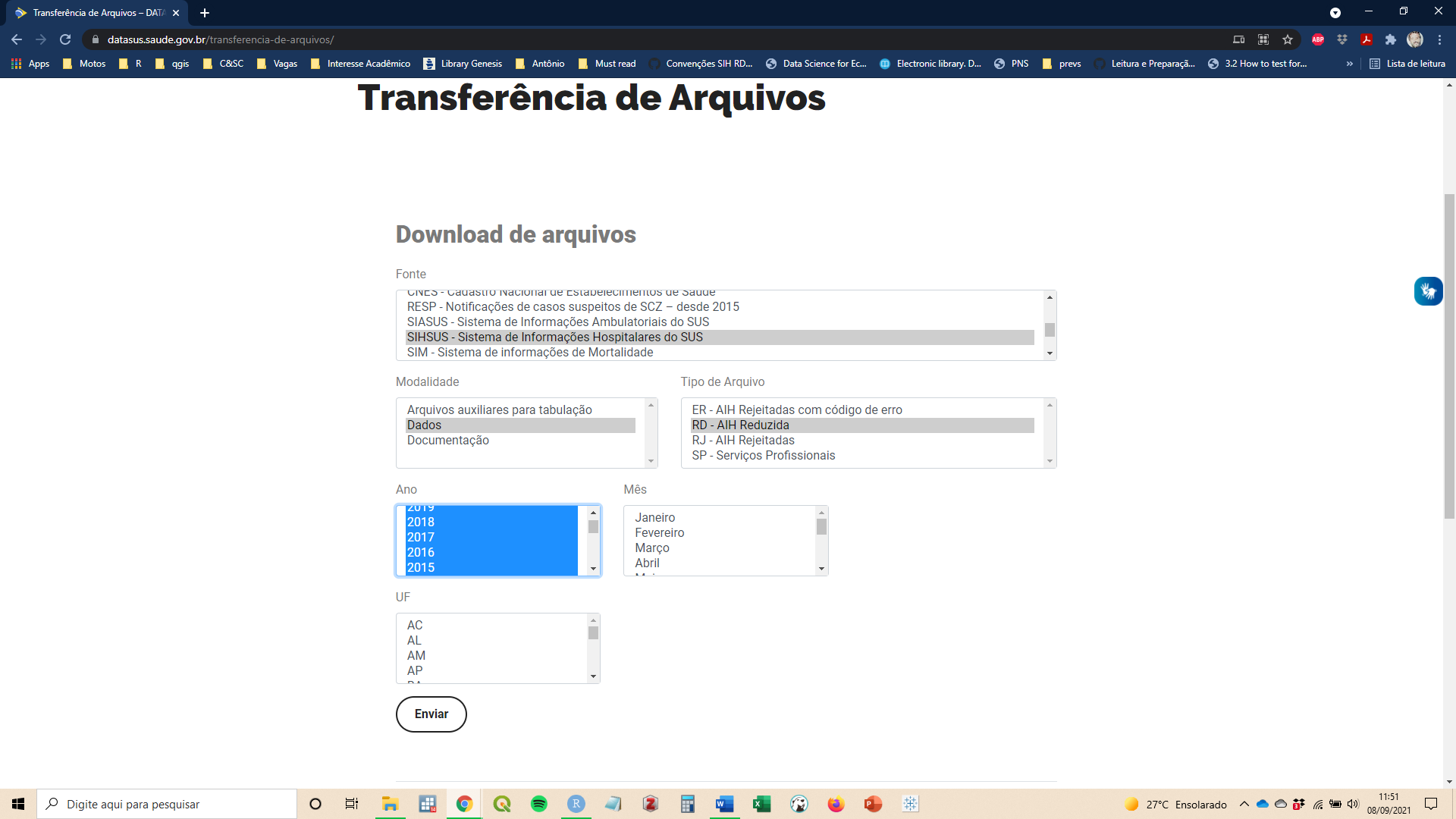


1. In the box named ‘Mês’, select the month or set of months of interest.


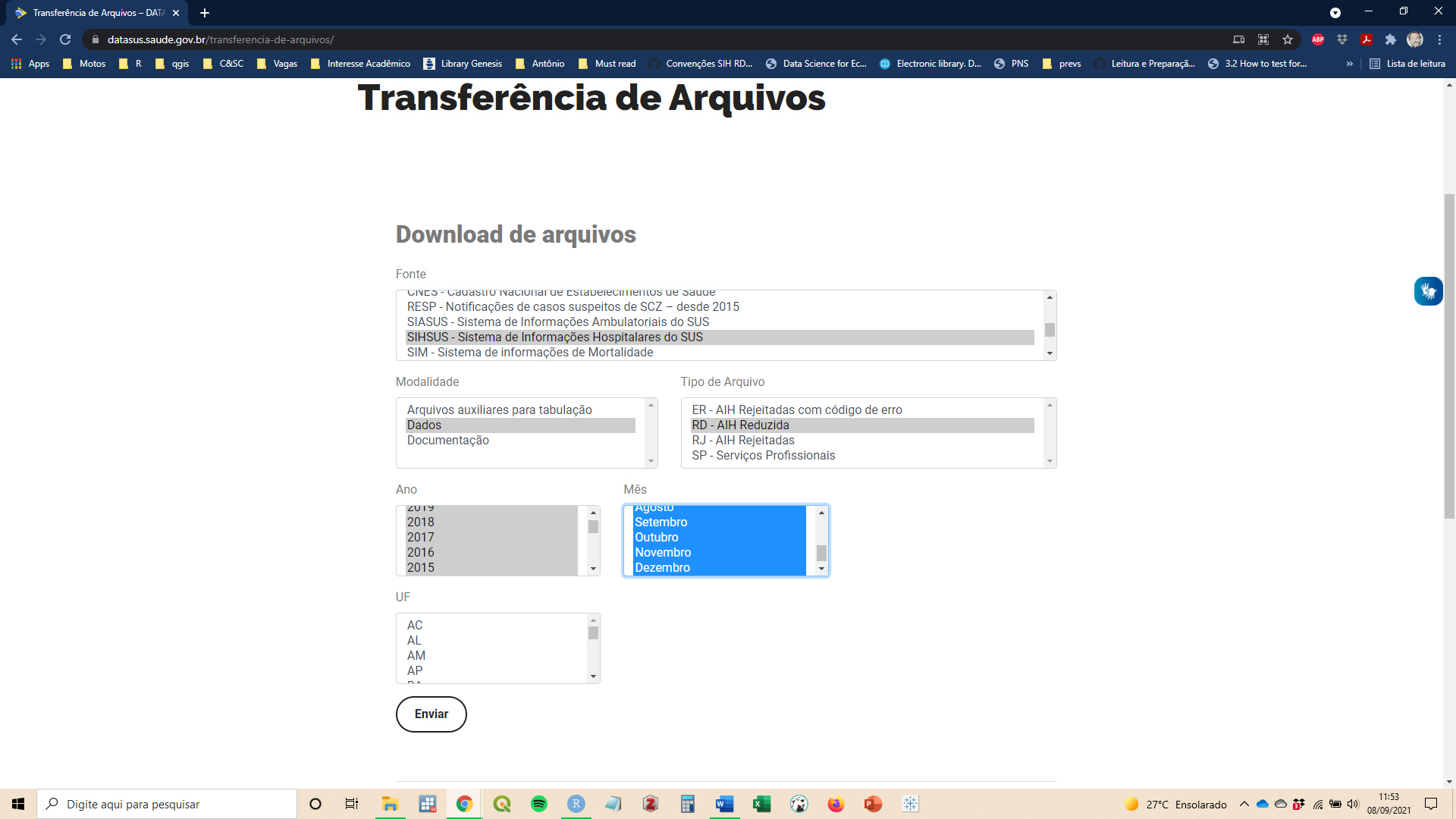


1. In the last box, named UF, choose the Brazilian state of interest. Belo Horizonte is located in Minas Gerais State, ‘MG’. Then, select ‘Enviar’.


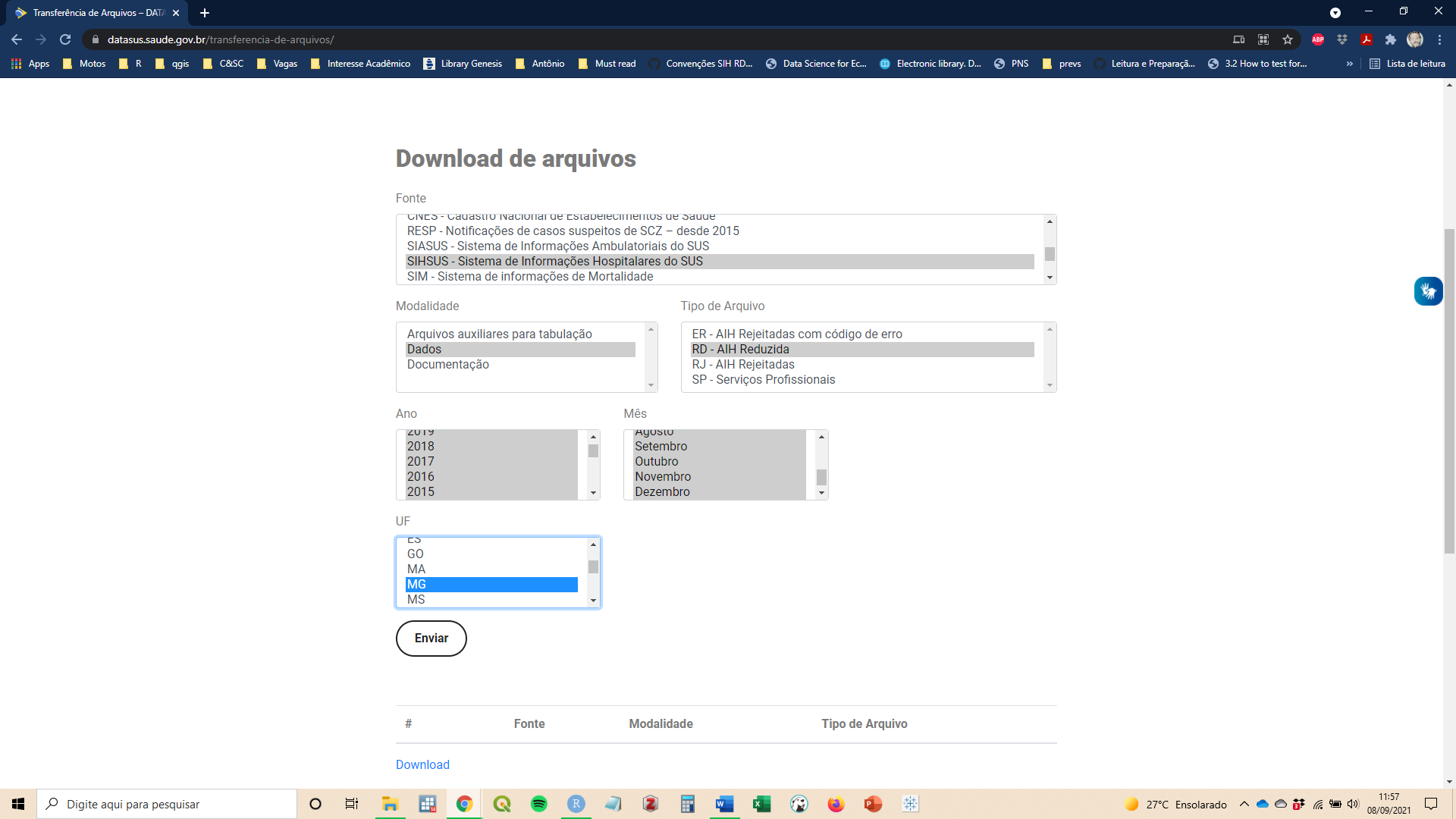


1. The selected files will be listed. At the bottom of the list you will see the download button. Files are named as ‘RD’+name of the STATE +year(YY)+month(MM)+’.dbc’


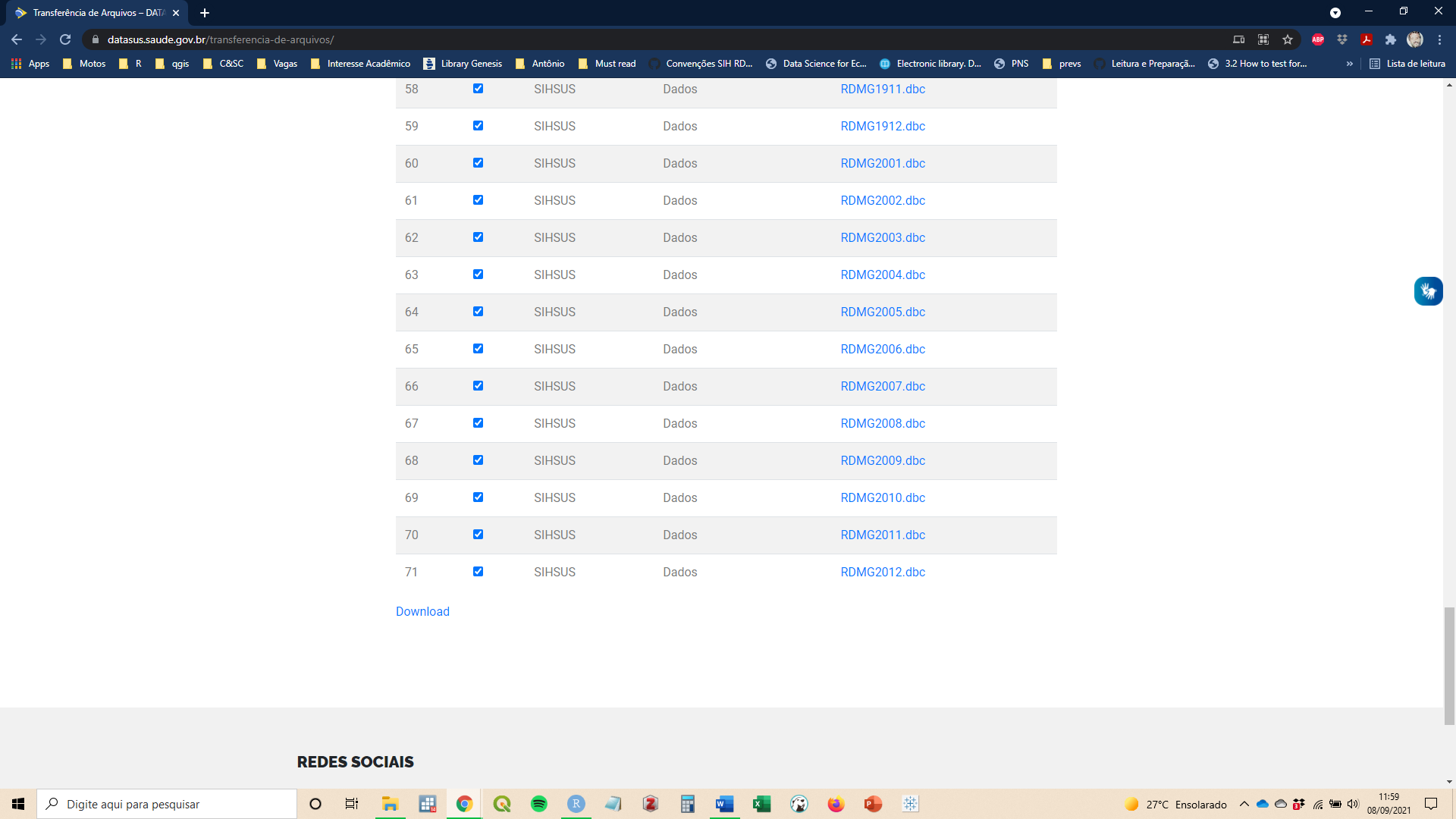

Supplement: S1 Text — (DOCX) [file pgph.0000054.s008.docx]
