# Supplementary material for: The impact of COVID-19 pandemic course in the number and severity of hospitalizations for other natural causes in a large urban center in Brazil
Source: PLOS Glob Public Health. 2021 Dec 20;1(12):e0000054. doi: 10.1371/journal.pgph.0000054 (PMC10021898; doi:10.1371/journal.pgph.0000054)
Supplement: Attachment — Submitted filename: RESPONSE TO REVIEWERS_Submitted.docx [file pgph.0000054.s009.docx]

**RESPONSE TO REVIEWERS**

**Ref:** The impact of COVID-19 pandemic course in the number and severity of hospitalizations for other natural causes in a large urban center in Brazil.

**Dear Editors,**

Please find below the answers to the reviewers’ comments. We would like to thank the careful reading and the comments that certainly improved the manuscript. We hope our answers and comprehensive text modification made this manuscript suitable publication in Plos Global Public Health. We are also available for further clarifications, if necessary.

Best regards,

Luisa Brant, on behalf of authors.

**REVIEWER #1**

**1. Conclusions are partly supported by the data. The aim of this study is to highlight the COVID-19 pandemic impact upon the number and severity of hospitalisation in public hospital, by non-COVID 19 natural causes, in Belo Horizonte city in 2020. Nevertheless, some relevant data could be presented and discussed, to avoid possible confusion biases, as the number of bed available in public hospitals for non COVID-19 hospitalisations, duration of hospital stay in 2015-2019 versus 2020 and number of in-patients' COVID-19 tests carried out if any. More explanation about the efficiency of public health policies deployed during this 2020 pandemic episode should be of interest (i.e. Mobile Emergency Medical Service). The economic characteristic of the population attending public health hospitals in Belo Horizonte city could be presented since introduction as a part of context. In the same way, the decrease of hospitalisation during slow-down measures could be due to a loss of income in a part of the population (day labourers/informal workers).**

**ANSWER:** We would like to thank for the comments, which will make the manuscript clearer. We have added information in the manuscript to better contextualize the healthcare system situation in Belo Horizonte and also the population that uses public hospitals in the city. In the sake of clarification, we changed the order of answers, as can be seen below.

*REVIEWER 1: “The economic characteristic of the population attending public health hospitals in Belo Horizonte city could be presented since introduction as a part of context. In the same way, the decrease of hospitalisation during slow-down measures could be due to a loss of income in a part of the population (day labourers/informal workers)”.*

In Methods, we have added the item “Population” to better describe the Brazilian health system and the population attended by it. The Brazilian Unified Health System (*Sistema Único de Saúde*, SUS) is funded by the government, with three ethical principles: universality of access, equity, and comprehensive care. Its main organizational features are decentralization, with emphasis on the municipal level, and regionalization of health services, so that in a defined area services are built in a network of growing complexity, from primary care to tertiary care.^1^ In Belo Horizonte, 50% of the population is dependent on SUS, and as such, are the users of public hospitals.^2^ In general, the SUS-dependent population is more socially vulnerable, have less education and income, but because healthcare is free of charge, loss of income during the pandemic cannot explain a change in the pattern of hospitalizations.

**Methods, line 88-97.** In Brazil, the public health system (Unified Health System, *Sistema Único de Saúde*, SUS) is constitutionally designed to provide the health as a "right for all" and a "duty of the state",^1^ and as such public hospitalizations are free of charge for all Brazilian citizens, although 50% of Belo Horizonte’s residents – usually those with higher socioeconomic conditions – have private health insurances and use private hospitals.^2^ In Belo Horizonte, the public health system infrastructure is well organized compared to the rest of the country – the city has one of the best quality primary health care program,^3^ and an organized mobile medical services, though inequalities in access to care cannot be excluded.^4^

1. Ribeiro ALP, Duncan BB, Brant LC, Lotufo PA, Mill JG, Barreto SM. Cardiovascular health in Brazil: trends and perspectives. Circulation. 2016;133(4): 422-33. doi: 10.1161/CIRCULATIONAHA.114.008727.
2. Agência Nacional de Saúde Suplementar (Brazil) [Internet]. Brasília (DF): Agência Nacional de Saúde Suplementar; 2021 [cited 2021 Jun 23]. Available from <https://www.gov.br/ans/pt-br>.
3. Guedes MBOG, Assis SJC, Sanchis GJB, Araujo DN, Oliveira AGRDC, Lopes JM. COVID-19 in Brazilian cities: impact of social determinants, coverage and quality of primary health care. PLoS One. 2021;16(9): e0257347. doi: 10.1371/journal.pone.0257347.
4. Guimarães NS, Carvalho TML, Machado-Pinto J, Lage R, Bernardes RM, Peres ASS, et al. Increased home death due to cardiopulmonary arrest in times of COVID-19 pandemic. Arq Bras Cardiol. 2021;116(2): 266-71. doi: 10.36660/abc.20200547.

*REVIEWER 1: ““Nevertheless, some relevant data could be presented and discussed, to avoid possible confusion biases, as the number of bed available in public hospitals for non COVID-19 hospitalisations, duration of hospital stay in 2015-2019 versus 2020 and number of in-patients' COVID-19 tests carried out if any. More explanation about the efficiency of public health policies deployed during this 2020 pandemic episode should be of interest (i.e. Mobile Emergency Medical Service).”*

In Belo Horizonte, the SUS infrastructure is well organized compared to the rest of the country – the city has one of the best quality primary health care program,^1^ and an organized mobile medical services, which indeed monitors the attended cases daily.^2^ In an article published in early 2021, a greater number of out-of-hospital cardiac arrest was reported in 2020, compared to 2019 and 2018,^3^ what was also seen in other places where hospitalizations for non-COVID causes declined,^4^ reinforcing our findings.

Regarding the public hospital’s organization during the COVID-19 pandemics in Belo Horizonte, four important features must be highlighted: 1) the Belo Horizonte health department monitors the daily demand and supply of total and ICU hospital beds, and increased the number according to the greater demands during the pandemics,^2^ as can be observed in the graphs below, which was added to the manuscript as **Supplementary Methods 1**. As such, an early hospital collapse was avoided due to adaptation of the health system, but we cannot separate from the information we have, which beds were dedicated to non-COVID-19 causes.

1. Guedes MBOG, Assis SJC, Sanchis GJB, Araujo DN, Oliveira AGRDC, Lopes JM. COVID-19 in Brazilian cities: impact of social determinants, coverage and quality of primary health care. PLoS One. 2021;16(9): e0257347. doi: 10.1371/journal.pone.0257347.

2. Belo Horizonte (MG). Coronavírus [Internet]. Belo Horizonte: Prefeitura de Belo Horizonte; 2021 [cited 2021 Jun 23]. Available from: <https://prefeitura.pbh.gov.br/saude/coronavirus>
3. Guimarães NS, Carvalho TML, Machado-Pinto J, Lage R, Bernardes RM, Peres ASS, et al. Increased home death due to cardiopulmonary arrest in times of COVID-19 pandemic. Arq Bras Cardiol. 2021;116(2): 266-71. doi: 10.36660/abc.20200547.
4. De Filippo O, D'Ascenzo F, Angelini F, Bocchino PP, Conrotto F, Saglietto A, et al. Reduced rate of hospital admissions for ACS during Covid-19 outbreak in Northern Italy. N Engl J Med. 2020;383(1): 88-9. doi: 10.1056/NEJMc2009166.

**Methods, line 102-104**: Through this surveillance, the municipality was able to prevent an early hospital collapse due to adaptation of the health system, creating new hospital and ICU beds as can be seen in **Supplementary Methods 1.**

**Supplementary Methods 1.** Number of hospital and intensive care unit beds in Belo Horizonte city from 2015 to 2020.

2) Comparing to the mean of hospitalizations and ICU utilization from 2015-2019, we can see in the novel supplementary files pasted below that the number of hospitalizations were rising from 2015 to 2019 (**Supplementary Figures 1 & 2**). However, in 2020, we observed a steep decline in this number, depicting the changes in trends. For ICU utilization, we also had a rising trend. However, in 2020, although hospitalizations declined in the whole pandemics period, ICU utilization declined in the first phase of the pandemics (in parallel with decreasing number of hospitalizations), but were still higher than the mean in the second phase of the pandemics onwards;

**Results, line 251-254:** In fact, **Supplementary Figures 1 and 2** reveal that the number of hospitalizations and ICU admissions were progressively rising in the city from 2015 to 2019, with a change in this rising trend to a decline when the pandemics started.

**Supplementary Figure 1.** Number of hospitalizations for non-COVID-19 natural causes in Belo Horizonte from 2015 to 2020.

**Supplementary Figure 2.** Number of Intensive Care Unit admissions for non-COVID-19 natural causes in Belo Horizonte from 2015 to 2020.

3) Duration of hospitalizations for non-COVID causes did not change in the pandemics period (**Supplementary Table 1**). This information has been added to the text. We hypothesized that this could be the result of two opposite trends: greater clinical severity, but an urge from clinicians to discharge patients to minimize risk of contracting COVID-19.

We added this information to the text:

**Methods, line 142-143:** The duration of hospitalizations were also analyzed for all groups of causes.

**Results, lines 189-191:** This substantial reduction in hospitalizations occurred with a concurrent maintenance in the duration of hospital stays for non-COVID-19 causes (**Supplementary Table 1**).

**Discussion, line 329-333:** Longer hospitalizations may also reduce the number of admissions, however duration of hospitalizations for non-COVID causes did not change in the pandemics period. We hypothesized that this could be the result of two opposite trends: greater clinical severity, but an urge from clinicians to discharge patients to minimize risk of contracting COVID-19.

4) Regarding COVID-19 in-hospital testing: although not widely available in the community setting, in hospitals all patients who received the diagnosis of COVID-19 had to have a positive RT-PCR or serology for COVID-19 for of his/her hospitalization be billed and payed, and this was checked by auditors. Because hospitalizations for COVID-19 payed more than other clinical hospitalizations, if COVID-19 was diagnosed, this was the chosen ICD-10 code to be assigned to the hospitalization. The described workflow was in place since February/2020

To the manuscript, we added the following sentences for clarification. If further information need to be added to the manuscript, we would be happy to do so.

**Methods, line 127-137**: During quality checks, this code has not been identified in hospitalizations from the previous 5 years. COVID-19 ICD-10 code could only be assigned to hospitalizations in which patients had a positive diagnostic test, either RT-PCR or serology, that should have been requested to all symptomatic patients upon hospitalization. The presence of a positive COVID-19 test was checked by auditors to avoid miscoding, particularly because COVID-19 hospitalizations paid more than other clinical hospitalizations.^1^ As each hospitalization in SIH is assigned only one code, if a positive test was present, a COVID-19 code was assigned, irrespective of comorbidities. Importantly, although there was a shortage of COVID-19 diagnostic tests in Belo Horizonte’s community setting, these tests were widely available in hospitals.

1.	Minas Gerais (Brazil). Secretaria de Estado de Saúde. Plano Estadual de Contingência para Emergência em Saúde: infecção humana pelo SARS-CoV-2 (doença pelo coronavirus – COVID-2019). Belo Horizonte: Secretaria de Estado de Saúde; 2020 [cited 2021 Jun 23]. Available from: <https://coronavirus.saude.mg.gov.br/images/2021/09/Plano_de_Contingencia_-_Revisao_3_-_03_09_2021.pdf>

**2. Data need to be presented in a more rigorous way: some discrepancies exist in the data presented in Results (lines 154-155) and Table 1: hospitalisation for natural causes and hospitalisation for non-COVID-19 natural causes are respectively 59,397 and 54,722 versus 73,690 and 69,015.**

**ANSWER:** Thank you for the detailed review. We comprehensively modified the Results section (see item 4, below). Unfortunately, due to high demands of work in COVID-19 times, and to timely analyse data from the pandemic to make them useful for health policy planning, we sent you preliminary tables and figures that have now been updated and checked for consistency. We apologize for that.

**Lines 185-282: Results** section has been updated to the new format of tables and figures presentation. Please refer to manuscript.

**3. Some sentences could be written in a more intelligible way (lines 60-64; 162-165; 279-282).**

**ANSWER:** We have corrected the sentences, as suggested. They have been marked in the manuscript in red.

**4. Figures could be clarified: only one variable by figure, allowing comparison between ICD-10 categories and corresponding units added.**

**ANSWER:** We updated the figures, tables, and text from the Results section to present the results according to your suggestion. As such, one variable (hospitalization, ICU utilization, and in-hospital deaths) was explored in each new figure, allowing comparison between ICD-10 categories.

**5. At the time of review, data were not available on the website** [**http://www2.datasus.gov.br/DATASUS/index.php?area=0901&item=1&acao=25**](about:blank) **are not available : "Erro no acesso à página".**

**ANSWER:** We regret the initially suggested link has been modified after the first draft was sent. We have updated the link and added a supplementary tutorial to reach the files.

The text was modified to:

**Methods, line 181-184:** The data that support the findings of this study are publicly available at [https://datasus.saude.gov.br/transferencia-de-arquivos/](about:blank), and the steps to reach the data are described in a tutorial (Supplementary File 1)

**REVIEWER #2**

**Thank you for submitting this manuscript. The topic is of relevance to a public health audience and in particular to health policy makers. I have a few preliminary concerns which would need to be addressed before reaching a final conclusion about the main statements brought by the authors in the results and discussion sections.**

Thank you for the encouraging comments. We hope to have clarified our statements in this revised version, and would be happy to provide further clarifications, if necessary.

**1) My first concern is about the possible systematic underreporting of all hospital admissions, regardless of the cause, in the context of COVID19 epidemic. This is briefly addressed in the discussion by the authors but in my opinion this could be a major bias that affects the message of this work. Do the authors have access to another source of data that would help estimating this systematic underreporting?**

**ANSWER**: Because the source of data of the present analysis is the administrative database used for payment purposes, there is virtually no underreporting. There could be reporting delays, although previous quality analysis revealed that in 4 months, more than 90% of hospitalizations were already included (see Supplementary Methods 2).The following sentences were added to the methods to clarify the completeness of the dataset, as has the table with quality checks in **Supplementary Methods 2**.

**Methods, line 110-111:** The SIH database is an administrative database for public hospitalizations, which is also used for hospital payment, resulting in virtually no underreporting.

**Methods, line 119-121:** The decision to exclude the last EW of the years studied was based in the date of data extraction, because previous quality checks of the data revealed that on average 90% of hospitalizations were processed within a 3 months-period after the date of hospitalization. This reporting delay between hospitalization and processing month can be seen in **Supplementary Methods 2.**

**2) My second concern is about a possible first wave of COVID19 from EW0 to EW15. How can the peak observed between EW0 and EW15, in particular in all causes ICU admissions, be explained? My understanding is that the first case of COVID19 in Belo Horizonte was reported in EW9, however can the possibility of a first wave of COVID19 between EW0 and EW15 be ruled out? Has this been investigated? What alternative hypotheses can be given to explain this rise in all-causes ICU admissions?**

**ANSWER:** We believe there was no first wave between EW 0-15 in Belo Horizonte for two reasons. First, Belo Horizonte was already warned by COVID-19 spread in other parts of the world and country, and COVID-19 tests, although not widely available in the community setting, were available for in-hospital diagnosis. An analysis made by Guedes *et al* about COVID-19 incidence in the 27 Brazilian capital cities revealed that Belo Horizonte had the fourth lowest incidence rates in the country in the first 26^th^ EW, 2020.^1^ Moreover, although not in the public system, Nascimento and cols showed that the largest private medical system in Belo Horizonte, which covers 1.2 million individuals (45% of the population), had a prepared telemedicine support system designed in December, 2019,^2^ reflecting that the healthcare workers in the city were aware of the COVID-19 spread.

Second and most important, our explanation for more hospitalizations, particularly ICU admissions from EW 0-15, is that there was a rising trend in hospitalizations and ICU admissions from 2015 to 2019 that was actually maintained in the beginning of 2020, until the COVID-19 pandemics changed the trend. This can be seen in the added **Supplementary Figures 1 & 2**, which are also pasted below. Particularly for ICU admissions, we observe that the pattern follows that of 2019, with a decline just before EW 10, due to the impact of the pandemics. We acknowledge that we cannot completely rule out missed COVID-19 diagnosis, but there is also no reason to believe there was a missing COVID-19 wave in the city between EW 0-15.

1. Guedes MBOG, Assis SJC, Sanchis GJB, Araujo DN, Oliveira AGRDC, Lopes JM. COVID-19 in Brazilian cities: impact of social determinants, coverage and quality of primary health care. PLoS One. 2021;16(9): e0257347. doi: 10.1371/journal.pone.0257347.
2. Nascimento BR, Brant LC, Castro ACT, Froes LEV, Ribeiro ALP, Cruz LV, et al. Impact of a large-scale telemedicine network on emergency visits and hospital admissions during the coronavirus disease 2019 pandemic in Brazil: Data from the UNIMED-BH system. J Telemed Telecare. 2020:1357633X20969529. doi: 10.1177/1357633X20969529.

**Supplementary Figure 2.** Number of hospitalizations for non-COVID-19 natural causes in Belo Horizonte from 2015 to 2020.

**Supplementary Figure 2.** Number of Intensive Care Unit Admission for non-COVID-19 natural causes in Belo Horizonte from 2015 to 2020.

To better address the issue, we added the following sentences to the methods, results and discussion, in addition to the 2 figures above:

**Methods, line 144-146:** The first COVID-19 diagnosis in Belo Horizonte occurred in EW 9, and the dates of the most important health policies in the pandemic course in the city were marked in the figures.

**Results, line 251-254:** In fact, **Supplementary Figures 1 and 2** reveal that the number of hospitalizations and ICU admissions were progressively rising in the city from 2015 to 2019, with a change in this rising trend to a decline when the pandemics started.

**3) My third concern results from the second: if a first wave of COVID19 gone undetected is plausible then my conclusion is that there was a significant underreporting of cases of COVID19 until coding practices were well established (either because of increased professional awareness, improved availability of specific diagnosis, etc.). This seems to be the case only after EW20 when the trend of COVID19-related hospital and ICU admissions actually followed that of all-causes, after a delay of about one month (the steep rise in hospital admissions and ICU is visible from EW15-EW17). For this reason, I would only draw conclusions on the differences between COVID19 and non-COVID19 related causes of hospitalizations after EW20. Conclusions on all causes remain valid for the whole period.**

**Answer:** As stated above, we cannot rule out missed COVID-19 diagnosis in the beginning of the pandemic, however we do not think a first undetected wave of COVID-19 occurred in Belo Horizonte from EW 0-15. Our explanation for the concurrent rise in COVID-19 hospitalizations (Figure 1, red line) and natural causes hospitalizations (Figure 1, blue line) is because what we named “Natural Causes” actually includes the COVID-19 hospitalizations. We made that clear in **Figure 1** and in the discussion, as described above.

**4) Many statistical tests were conducted but I do not have enough information to conclude about their relevance, please refer to my comments for the Methods and Results sections. The main message of the manuscript would benefit from a more concise reporting of the results, especially for the four categories of non-covid causes of admissions (Table 1 and Figures 2-5).**

**Answer:** The relevance of statistical tests was strengthened. We added the lowest and highest values to all variables in the tables and a graphic presentation of this interval in all figures. Moreover, we have changed, as suggested by Reviewer 1, the strategy for showing our results. The Figures were reconstructed for each variable (Hospital admissions, ICU admissions, in-hospital deaths). We hope that these changes make the results section clearer and more concise.

**5) Introduction: The introduction is clear and concise, the context is well described, the objectives and underlying hypotheses well exposed. The topic is indeed highly relevant. I have no specific comments about this section.**

**Answer*:*** Thank you for the encouraging comment.

6**) Methods**

**6.1) More information would be welcome regarding how patients were classified in Belo Horizonte with the ICD coding. Could a patient be classified with several different ICD codes, if so, which one was chosen and according to which rule? For example would a patient with cardiac failure aggravated by COVID19 admitted in cardiology ICU by classified?**

**Answer:** In Brazil, each patient has only one ICD-10 code for each hospitalization. If COVID-19 was diagnosed during the hospitalization, the ICD code for COVID-19 was assigned to the patient. Though not widely available in the community setting, all symptomatic patients were tested in-hospitals with RT-PCR or serology (IgM and IgG) and if positive, the patient received the COVID-19 diagnosis. Because hospitalizations for COVID-19 payed more than other clinical hospitalizations, if COVID-19 was diagnosed, this was the chosen ICD-10 code to be assigned to the hospitalization, which were checked by auditors for positive tests. ICD coding regarding other diseases rather than COVID-19 did not change during the pandemics. As such, patients with other chronic diseases that were hospitalized in the context of acute COVID-19 were considered as having COVID-19 and not counted for other diseases. We made this clearer in the Methods section.

**Methods, line 126-137:** Hospitalizations were considered by COVID-19 if the B34.2 ICD-10 code were used. In quality control analysis, this code has not been identified in hospitalizations from the previous 5 years. COVID-19 ICD-10 code could only be assigned to hospitalizations in which patients had a positive diagnostic test, either RT-PCR or serology, that should have been requested to all symptomatic patients upon hospitalization.^1,2^ The presence of a positive COVID-19 test was checked by auditors to avoid miscoding, particularly because COVID-19 hospitalizations paid more than other clinical hospitalizations. As only one code is assigned to each hospitalization, if a positive test was present, a COVID-19 code was assigned irrespective of comorbidities. Importantly, although there was a shortage of COVID-19 diagnostic tests in Belo Horizonte’s community setting, these tests were widely available in hospitals.

1. Minas Gerais (Brazil). Secretaria de Estado de Saúde. Plano Estadual de Contingência para Emergência em Saúde: infecção humana pelo SARS-CoV-2 (doença pelo coronavirus – COVID-2019). Belo Horizonte: Secretaria de Estado de Saúde; 2020 [cited 2021 Jun 23]. Available from: <https://coronavirus.saude.mg.gov.br/images/2021/09/Plano_de_Contingencia_-_Revisao_3_-_03_09_2021.pdf>
2. Brazil. Ministéio da Saúde. Portaria n^o^ 245, de 24 de março de 2020. Inclui procedimento na Tabela de Procedimentos, Medicamentos, Órteses, Próteses e Materiais Especiais (OPM) do Sistema Único de Saúde (SUS), para atendimento exclusivo de pacientes com diagnóstico de infecção pelo COVID-19 [...]. Brasília (DF), Diário Oficial da União. 2020 Apr 30. [cited 2021 Jun 23]. Available from: <https://www.in.gov.br/en/web/dou/-/portaria-n-245-de-24-de-marco-de-2020-*-254675204>

**6.2) Was the coding procedure consistent? How were these practices impacted by the epidemic? Did they improve over time?**

**Answer:** Coding procedures for COVID-19 may indeed be susceptible to learning effects. However, because in Belo Horizonte a positive RT-PCR or serology was necessary to code the patient’s hospitalization as COVID-19, the diagnosis for hospitalizations were objective, and the learning curve could have been related to recognizing symptoms of the disease and requesting the COVID-19 test by physicians. This may have happened, in which case a patient with COVID-19 would receive an alternative diagnosis and the real decline in non-COVID-19 hospitalizations would be even greater, reinforcing our results. For other diseases, the coding procedures did not change during the pandemics period. We have added this information in:

**Methods, line 126-137:** Hospitalizations were considered by COVID-19 if the B34.2 ICD-10 code were used. In quality control analysis, this code has not been identified in hospitalizations from the previous 5 years. COVID-19 ICD-10 code could only be assigned to hospitalizations in which patients had a positive diagnostic test, either RT-PCR or serology, that should have been requested to all symptomatic patients upon hospitalization. The presence of a positive COVID-19 test was checked by auditors to avoid miscoding, particularly because COVID-19 hospitalizations paid more than other clinical hospitalizations. As only one code is assigned to each hospitalization, if a positive test was present, a COVID-19 code was assigned irrespective of comorbidities. Importantly, although there was a shortage of COVID-19 diagnostic tests in Belo Horizonte’s community setting, these tests were widely available in hospitals.

**Discussion, lines 389-395:** About miscoding, first, coding procedures for non-COVID-19 hospitalizations – the focus of our analysis – did not change during the period. Because COVID-19 could only be coded in the presence of a positive diagnostic test, the main potential bias would be to have hospitalized patients with COVID-19 that would receive an alternative diagnosis in case a COVID-19 diagnostic test was not requested. In this scenario, the real decline in non-COVID-19 hospitalizations would be even greater, reinforcing our results.

**6.3) How were cases of COVID-19 identified in Belo Horizonte in 2020? What diagnostic tests were available and when? Was the case definition consistent over time?**

**ANSWER:** Unfortunately, the lack of tests in the country certainly resulted in underreporting of cases in the community, but not of in-hospital cases. Symptomatic individual admitted to a public hospitals in Belo Horizonte were test by RT-PCR or serology for SARSCoV2. These tests were available from February/2020. We have added the following sentence to the manuscript.

**Methods, line 126-137:** Hospitalizations were considered by COVID-19 if the B34.2 ICD-10 code were used. In quality control analysis, this code has not been identified in hospitalizations from the previous 5 years. COVID-19 ICD-10 code could only be assigned to hospitalizations in which patients had a positive diagnostic test, either RT-PCR or serology, that should have been requested to all symptomatic patients upon hospitalization. The presence of a positive COVID-19 test was checked by auditors to avoid miscoding, particularly because COVID-19 hospitalizations paid more than other clinical hospitalizations. As only one code is assigned to each hospitalization, if a positive test was present, a COVID-19 code was assigned irrespective of comorbidities. Importantly, although there was a shortage of COVID-19 diagnostic tests in Belo Horizonte’s community setting, these tests were widely available in hospitals.

**6.4) Line 103: Could the authors explain how this quality checks were performed for the previous years (how was the 90% figure determined, with what reference data) and whether this was also performed for 2020? This could help give insight on a possible systematic underreporting in 2020.**

**Answer:** The information system provides information about the date when the hospitalization occurred and the date (month) in which the information was processed. We constructed a cross table, new **Supplementary Table 1**, with the proportion of hospitalizations by processing month and admission month. This table allowed us to check the proportion of hospital admissions from a given month that were processed in the following months. In general, 90% of hospital admissions from a given month were processed in a three-month period. We did a preliminary analysis of 2020 data and observed a similar pattern. We have changed the manuscript to make this issue clearer, and have added a supplementary table to illustrate the information in the Methods section.

**Methods, Line 116-121:** The decision to exclude the last EW of the years studied was based in the date of data extraction, because previous quality checks of the data revealed that on average 90% of hospitalizations were processed within a 3 months-period after the date of hospitalization. This reporting delay between hospitalization and processing month can be seen in **Supplementary Methods 2.**

**Supplementary Methods 2:** Hospital Admission Month and Hospitalization Processing Month in Belo Horizonte, from January 2019 to May 2020.

**6.5) Line 129: Are ICU admissions included in the total hospitalization admissions?**

**Answer:** Yes. We modified the following sentence to better specify this issue in the Methods:

**Methods, Line 124:** We examined by EW the following variables: number of hospitalizations and **number and percent of hospitalizations resulting in intensive care (ICU admissions) or in death (in-hospital deaths).**

**6.6) Lines 138-140: Wilcoxon rank sum test was used. Can the authors confirm the following: for each outcome variable the statistical test compared a single estimate (for year 2020) to the distribution of five estimates (for each year 2015 to 2019)?**

**Answer**: We compared 2020 estimates with a single estimate: the observed mean in the 2015-2019 period. We did not compare 2020 estimates with each specific year (2015, 2016, 2017, 2018, 2019).

We made it clearer in the Methods:

**Methods, line 158-161:** In **Figures 1 to 4**, the five-week moving averages of the studied variables from January to November, 2020, were compared to a single estimate: the five-week moving averages of the same variables for the previous period (January 2015 to November 2019), for each group of diseases.

**6.7) Line 141: Referring to my concern of a possible unreported wave of COVID19 between EW0 and EW15 and possible associated lack of appropriate coding of COVID19 cases: I would suggest to begin the analysis at EW0 for all natural causes, or EW20 when distinguishing COVID19-related and non-COVID19-related causes.**

**ANSWER:** Because of the reasons exposed in our answer to question 2 above, we think that the analysis could be maintained considering the period previously analysed.

**6.8) Line 142: How was the Benjamini & Hochberg correction performed? If I am not mistaken, a total of 180 tests were performed:
-30 for the main results in Table 1 (5 outcomes * 6 categories of diseases)
-90 tests for age-stratified results (5 outcomes * 6 categories of diseases * 3 categories of age)
-60 tests for sex-stratified results (5 outcomes * 6 categories of diseases * 2 categories of sex)
Was the BH correction brought to the total of 180 tests?**

**ANSWER:** We compared each 2020 estimate with the corresponding mean for the 2015-2019 period. After each Wilcoxon rank sum test was performed, we applied the Benjamini & Hochberg correction for each analysed category. We made changes in the manuscript to make that clearer.

**Methods, line 171-174:** We compared 2020 estimates with a single estimate: the observed mean in the 2015-2019 period. The resulting P-values were afterwards corrected using the Benjamini & Hochberg correction for multiple testing and were considered significant if p ≤ 0.05.

**7) Results:**

**7.1) Table 1 and supplementary tables: No information is provided about the fluctuation around the mean outcomes reported for 2015-2019. This would help interpret the differences and associated p-values more easily.**

**ANSWER:** The lower and upper values for the same EW between 2015-2019 were included in all tables and figures. Besides that, we included two supplementary figures (1 and 2) on the trends of hospitalizations and ICU admissions for 2015-2020, as already shown.

**Methods, line 161-165:** The lower and upper values for the same EW from 2015 to 2019 were also included in all tables and figures. To investigate trends hospitalizations and ICU admissions along the years 2015 to 2020, we graphically compared observed data from EW of each cited year to the mean, lower and upper limits of 2015-2019.

**7.2) Could the authors provide the maximum and minimum values for each outcome for 2015-2019, or a standard deviation?**

**ANSWER**: The new figures 2-4 show the interval between the maximum and minimum values for each EW from 2015 to 2019. This information has been added to the text.

**Methods, line 161-162:** The lower and upper values for the same EW from 2015 to 2019 were also included in all tables and figures.

**7.3) Line 165: The figures reported in the text do not match those shown in lines 4 and 5 of section 2 in Table 1.**

**ANSWER:** We replaced all tables and figures 2-5 (now 2-4) and checked the text.

**7.4) Line 191 and 194: change “ICU admissions” by “proportion of ICU admissions”**

**ANSWER:** Thank you, the Results section (lines 189-278) was rewritten in light of the new format of Tables and Figures.

**7.5) Lines 185, 186; 191, 193: The authors make several claims of statistical significance but no mention of confidence interval or p-values are made in the supplementary tables. Although I can assume that asterisks mean a p-value < 0.05, there is no such indication in the supplementary tables. Again, how was Benjamini-Hochberg correction applied?**

**ANSWER:** Thank you. Asterisks mean a p-value < 0.05, what was clarified in the tables. The intervals between the maximum and minimum values for each EW from 2015 to 2019 were added. Benjamini-Hochberg correction was applied as previously described for question 6.8. These information were added to the text, as previously described.

**7.6) Figures 1-5: Again, it is important to visualize the fluctuation around the mean for 2015-2019 to assess the extent of the differences reported for 2020. For 2015-2019, could the authors show on the figures the weekly maximum and minimum, or each year’s curve individually?**

**ANSWER:** Agreed. The information was added, as requested.

**7.7) In 2020, a steep decrease is observed for all natural causes after EW45. In the methods, the authors explain that data beyond EW48 was ignored; could this drop be attributed to a greater delay in reporting data for the end of year 2020 that affected EW45 to EW48 as well?**

**ANSWER:** Indeed, the delay on data collection was the reason we decided to analyse data until EW48. We cannot assure that data from EW 45-48 had a greater reporting delay than that from previous months. Although this is a possibility, which was added to the text, we did not see a change in the pattern of the processing months for hospital admission until May/2020, as shown in **Supplementary Table 1** (pasted above).

**Discussion, line 387-389:** However, due to the changes in routines during pandemic times, we cannot assure the pattern was the same in the end of 2020, and the decline in hospitalizations from EW 45 onwards may result from reporting delays.

**7.8) I find that the text and figures lack a consistent designation for each non-covid specific category; sometimes the ICD chapter is used, sometimes different designations (e.g. “diseases of the circulatory system” in Table 1, “Chapter IX” in Figure 5, “cardiovascular diseases” in Figure 5 legend). The manuscript would benefit from an improved consistency on that matter.**

**ANSWER:** Thank you, we standardized the designation by the diseases definition, instead of the ICD Chapter.

**7.9) The labeling of figure 3 and 4 is incoherent: the legends refer to neoplasms and respiratory diseases, respectively, whereas the figures themselves refer to chapter X (respiratory diseases) and II (neoplasms), respectively. Given the shape of the mean trend for 2015-2019 (seasonal in figure 3 and constant in figure 4), I guess that the information on the figures and correct and the legends are wrong.**

**ANSWER:** Thank you, we hope our new proposal is clearer.

**7.10) The multiplication of the figures for each specific cause, each with a very similar legend does not help understanding. I suggest simplifying the analysis and the presentation of the results by grouping the non-covid causes of admission in two categories: seasonal or communicable (respiratory and infectious diseases) vs. non-seasonal or non-communicable (cardiovascular diseases and neoplasms), based on the observation that each follow similar patterns as noted in the manuscript**.

**ANSWER:** We modified the figures as suggested by reviewer 1, but opted to maintain the grouping of causes, since neoplasms and CVD presented a different profile during the pandemics. We hope this new format makes the presentation of results clearer.

**7.11) Line 198: “beginning of the pandemic (EW11-21)” the first red line in the figures is set to EW9 whereas in the methods (line 120) it is mentioned that the first control phase started in EW11.**

**ANSWER:** The figures were updated and corrected.

**7.12) Line 198-199 “there was a reduction in the number of non-COVID-19 hospitalizations” On the EW11-EW21 there was almost no cases of COVID-19 reported. This statement should mention all causes of admissions.**

**ANSWER:** Agreed. We have changed all the Results section text, in light of our new proposal for tables and figures as suggested by Reviewer 1.

**7.13) Line 200-201: “which then rises steeply after the first reopening at EW21”. The increase seems to start earlier for all natural causes (week 15 for hospital admissions, week 17 for ICU and deaths). The rise is only seen in week 20 for COVID19, and is then possibly aggravated by the removal of control measures in week 21. Beyond week 21 we can see clearly a difference between the dynamics of COVID19 and non-COVID19 causes, likely due to a better classification of cases in an epidemic context.**

**ANSWER:** We have changed the text for the Results section. However, our explanation for the findings are: the increase in hospitalizations that starts earlier than EW20 are from non-COVID-19 causes (green line for non-COVID-19 causes rises concurrently with the blue line “Natural causes, including COVID-19”), and not due to undiagnosed COVID-19, as explained previously. In EW 15, other cities in Brazil such as Manaus were already in health collapse, so there is no reason to believe there was substantial in-hospitals’ underdiagnosis in Belo Horizonte, where tests were available. We understand this was a result of the reduction in the fear of the population in searching for health facilities, as the incidence of COVID-19 cases were still low in the city. Indeed, the green and blue curves only separate in EW 21, when the city reopened and COVID-19 spread faster.

**7.14) Lines 206-207: I would only consider this statement to hold true beyond EW21.**

**ANSWER:** We have changed the text for the Results section, but our potential explanations for these findings are described above. In our view, although in-hospital coding may have improved due to more precise recognition of symptoms, we do not believe that miscoding alone explains the patterns seen.

**8.8) Discussion:**

**The discussion is interesting and in agreement with the results. However some points would benefit from more caution and may need revision in the light the concerns exposed above**.

**8.1) The practice and the quality diagnosis and ICD classification of patients would merit more discussion, in the light of my preliminary comments.**

**ANSWER:** We agree and have clarified this issue in the sentences in the text already cited in the Methods section. In the discussion section, he have added the following sentences**:**

**Discussion, lines 387-395:** However, due to the changes in routines during pandemic times, we cannot assure the pattern was the same in the end of 2020, and the decline in hospitalizations from EW 45 onwards may result from reporting delays. About miscoding, first, coding procedures for non-COVID-19 hospitalizations – the focus of our analysis – did not change during the period. Because COVID-19 could only be coded in the presence of a positive diagnostic test, the main potential bias would be to have hospitalized patients with COVID-19 that would receive an alternative diagnosis in case a COVID-19 diagnostic test was not requested. In this scenario, the real decline in non-COVID-19 hospitalizations would be even greater, reinforcing our results.

**8.2) Could part of the decline of hospitalizations for specific causes could be explained by a coding artifact of admissions: the preferential coding of COVID19 cases over any underlying condition (eg: cardiac failure, respiratory failure, cancer etc.)?**

**ANSWER:** This was not the case because for a hospitalization to be coded as COVID-19, the patient had to have a positive COVID-19 test, and this information was audited. This information has been stated in the Methods section.

**Methods, line 128-131:** COVID-19 ICD-10 code could only be assigned to hospitalizations in which patients had a positive diagnostic test, either RT-PCR or serology, that should have been requested to symptomatic patients upon hospitalization.

**8.3) Line 314: I would be more cautious before interpreting the differences between men and women for a single outcome and a specific category of diseases, given the possible limits of the data.**

**ANSWER:** Agreed. Although this is a finding corroborated by consolidated knowledge regarding the disadvantage between sexes in CVD treatment, we have been more cautious in the discussion.

**Discussion, 337-341:** The analysis stratified by sex did not reveal consistent significant differences, with the exception of the higher proportional in-hospital mortality by CVD and respiratory diseases for women. As such, the COVID-19 pandemic may be further increasing the gap in treatment for cardiovascular diseases in women,^23^ although due to the limits of data after stratification, these findings must be interpreted with caution.

**8.4) Line 366: It seems that there were still quite some lack of information from EW40, explaining the drop at the end of year 2020 (see my comment in the results)**

**Answer**: We hope our previous explanations clarified this issue. We have added this hypothesis to the discussion.

**Discussion, Line 387-389:** However, due to the changes in routines during pandemic times, we cannot assure the pattern was the same in the end of 2020, and the decline in hospitalizations from EW 45 onwards may result from reporting delays.

8.5) Line 383: This final statement is quite welcome.

**Answer**: Thank you.
